# Identification of genes differentially expressed during prenatal development of skeletal muscle in two pig breeds differing in muscularity

**DOI:** 10.1186/1471-213X-7-109

**Published:** 2007-10-01

**Authors:** Eduard Muráni, Mária Murániová, Siriluck Ponsuksili, Karl Schellander, Klaus Wimmers

**Affiliations:** 1 Research Institute for the Biology of Farm Animals (FBN), Research Unit Molecular Biology, Wilhelm-Stahl-Allee 2, 18196 Dummerstorf, Germany; 2 Institute of Animal Science, Animal Breeding and Husbandry Group, University of Bonn, Endenicher Allee 15, 53115 Bonn, Germany; 3 Research Institute for the Biology of Farm Animals (FBN), Research Group Functional Genomics, 18196 Dummerstorf, Germany

## Abstract

**Background:**

Postnatal muscle growth is largely depending on the number and size of muscle fibers. The number of myofibers and to a large extent their metabolic and contractile properties, which also influence their size, are determined prenatally during the process of myogenesis. Hence identification of genes and their networks governing prenatal development of skeletal muscles will provide insight into the control of muscle growth and facilitate finding the source of its variation. So far most of the genes involved in myogenesis were identified by *in vitro *studies using gene targeting and transgenesis. Profiling of transcriptome changes during the myogenesis *in vivo *promises to obtain a more complete picture. In order to address this, we performed transcriptome profiling of prenatal skeletal muscle using differential display RT-PCR as on open system with the potential to detect novel transcripts. Seven key stages of myogenesis (days 14, 21, 35, 49, 63, 77 and 91 *post conception*) were studied in two breeds, Pietrain and Duroc, differing markedly in muscularity and muscle structure.

**Results:**

Eighty prominent cDNA fragments were sequenced, 43 showing stage-associated and 37 showing breed-associated differences in the expression, respectively. Out of the resulting 85 unique expressed sequence tags, EST, 52 could be assigned to known genes. The most frequent functional categories represented genes encoding myofibrillar proteins (8), genes involved in cell adhesion, cell-cell signaling and extracellular matrix synthesis/remodeling (8), genes regulating gene expression (8), and metabolism genes (8). Some of the EST that showed no identity to any known transcripts in the databases are located in introns of known genes and most likely represent novel exons (e.g. *HMGA2*). Expression of thirteen transcripts along with five reference genes was further analyzed by means of real-time quantitative PCR. Nine of the target transcripts showed higher than twofold differences in the expression between the two breeds (*GATA3*, *HMGA2*, *NRAP*, *SMC6L1*, *SPP1*, *RAB6IP2, TJP1 *and two EST).

**Conclusion:**

The present study revealed several genes and novel transcripts not previously associated with myogenesis and expands our knowledge of genetic factors operating during myogenesis. Genes that exhibited differences between the divergent breeds represent candidate genes for muscle growth and structure.

## Background

Prenatal development of skeletal muscle, myogenesis, is an ideal model to study cell determination and differentiation. Accordingly myogenesis has become an extensively studied process in model animals [1]. Because muscle regeneration resembles myogenesis the study of myogenesis provides basic knowledge for the development of therapies to treat human muscle disease [2]. In farm animal biology the interest in myogenesis is driven mainly by the perception that muscle growth potential is associated with muscle fiber number and muscle structure, i.e. with characteristics that are determined prenatally during myogenesis [3]. The relationship between muscle mass and muscle fiber number as well as muscle structure is well illustrated by the observation that domestic pigs selected for muscularity exhibit a higher number of myofibers and an increased proportion of fast twitch glycolytic fibers (the myofiber type with the largest cross sectional area in the pig) compared to their wild ancestor or unimproved breeds [3,4]. Morphological studies of myogenesis in the pig are scarce. Development of somites takes place approximately between day 14 and 22 of gestation. Around day 35 of gestation the formation of primary myotubes starts and proceeds until around day 60. The secondary population of myotubes appears around day 50 (45–54) on the surface of the primary myotubes, which they use as a scaffold. The number of secondary myotubes increases several fold until day 75. Afterwards the number increases only slightly. Around days 85–90 the fiber formation ceases and the total number of fibers is established. The process of maturation of the myotubes into myofibers is finished in the early postnatal period [5,6]. In the pig, primary myotubes are a minor constituent of muscle fibers. In adult muscle, the ratio of primary to secondary myotubes is about 1:20. However, both populations of myotubes significantly influence fiber number and consequently muscle size. The importance of both primary and secondary myotubes for muscle growth is underscored by the lower number of primary fibers and a lower secondary to primary fiber ratio in small compared to large pig breeds [7,8].

Knowledge of genetic and epigenetic factors that govern formation of both generations of myotubes will facilitate the understanding of the control of muscle growth and structure. Application of transcriptome profiling technologies largely enhances our knowledge of these factors and facilitates uncovering the myogenic gene networks. So far transcriptome profiling of myogenesis in livestock was performed using macroarrays [9,10] or application specific microarrays [11-13]. However, only a limited number of specified genes could be analyzed using these methodologies.

In the present study we employed a complementary methodology, differential display RT-PCR, as an open system that facilitates the identification and the analysis of yet unknown transcripts. We analyzed myogenesis in two breeds differing in muscularity and muscle structure: the Pietrain breed with higher muscularity, higher number of muscle fibers and a higher proportion of glycolytic muscle fibers and the Duroc breed with lower muscularity and higher proportion of slow twitch muscle fibers [4,14]. The aim of this study was the identification of genes and pathways whose expression profile during development of porcine skeletal muscle implicates their involvement in myogenesis. In addition genes that were found to be differentially expressed between Pietrain and Duroc represent candidate genes with potential impact on muscle growth and structure.

## Results

### Differential display RT-PCR profiling of the transcriptome during porcine myogenesis

Comparison of a total of 88 differential display profiles between stages and between breeds revealed 458 cDNA fragments differing in their presence and/or in their intensity. Out of these, 310 cDNA fragments showed similar stage-associated differences in the expression in both breeds and 148 fragments were differentially displayed between breeds. Though the essentially binary expression patterns and the inefficient and tedious cloning of the differentially displayed cDNA fragments hamper a global view of the expression changes and a comprehensive pathway analysis, we attempted to assign cDNA fragments to groups based on their expression patterns and to relate these to functional pathways. Stage-associated cDNA fragments were classified into five groups of patterns ('period' groups) according to their distribution and/or changes in the intensity between the stages. The first group is related to early development (period 1; either present only or apparently upregulated at 14 dpc and 21 dpc). The second and third groups are related to the first wave (period 2; either present only or apparently upregulated at 14 – 49 dpc) and the second wave (period 3; either present only or apparently upregulated at 49 – 91 dpc) of myogenesis respectively. The fourth group included fragments present at both first and the second wave of myogenesis (period 4; either present only or apparently upregulated at 21 – 91 dpc). The fifth group contained fragments present at all 7 stages that showed either steady up- or downregulation (period 5). In order to perform a similar grouping of the breed-associated cDNA fragments according to their temporal expression across stages one such fragment was called present at a particular stage if it was present in one of the two breeds (under the assumption that the absence in the other breed was due to lower expression level or a SNP in the priming site).

Reamplification, cloning and sequencing was successfully performed for 43 breed- and 37 stage-associated cDNA fragments (Tables 1 and 2). Two breed-associated and four stage-associated differential display bands were found to represent multiple (2–3) different cDNA fragments of nearly identical length. The sequences of the cDNA fragments bR21D1 and bR10D1 were embedded in the larger EST bS19C1 and qR10D1, respectively, thus in total 85 unique EST (44 breed- and 41 stage- associated) were generated [GenBank:DQ631863, EH792585–EH792670]. Homology search revealed that 45 EST represented known genes, 20 EST showed similarity to other anonymous EST/cDNA in the database, 3 EST showed similarity to genomic clones, 2 EST were homologous to mitochondrial DNA (genes) and 17 EST did not match any database entries. The *MALAT1 *gene was represented by two different EST representing cDNA fragments with similar expression patterns (qR24B1 and bS22B1#2; period 4). Taking advantage of the high homology between pig and human sequences, we could assign 8 EST similar to anonymous EST/cDNA and 2 EST with genome hits to gene loci by BLAST search of the human genome. In total 55 EST were assigned to 52 genes.

**Table 1:** Summary of EST showing stage-associated differential expression during porcine myogenesis

EST/Clone	Accession number	Identity^1^	Species	Accession number	Similarity	Function	Period group	Expression pattern^2^
bS9C3	EH792591	Syndecan 3 (SDC3)	Bovine	XM_870523	106/127 (83%)	cell adhesion, ECM	1	1-1-0-0-0-0-0
bS17A2	EH792585	Ribosomal protein L37a (RPL37A)	Canine	AY197363.1	146/172 (84%)	metabolism	1	0-1 > 1-1-1-1-1
bS24A4	EH792589	Glutamate dehydrogenase 1 (GLUD1)	Human	X67491.1	157/173 (90%)	metabolism	1	1-1-0-0-0-0-0
bS9B1	EH792590	EST	Porcine	BE235673	237/283 (83%)	other, unknown	1	1-0-0-0-0-0-0
bS17C2	EH792586	Unknown				other, unknown	1	0-1-0-0-0-0-0
bS17D3	EH792587	EST	Bovine	CB169367	146/169 (86%)	other, unknown	1	0-1-0-0-0-0-0
bS18D2	EH792588	Unknown				other, unknown	1	1-1-0-0-0-0-0
bS8D1	EH792602	ATG3 autophagy related 3 homolog (S. cerevisiae) (ATG3)	Human	NM_022488	132/146 (90%)	cell death, autophagy	2	0-1-1-0-0-0-0
bS9C4	EH792603	EST, [Insulin-like growth factor binding protein-like 1 (IGFBPL1)]	Porcine	CK465405	202/205 (98%)	cell proliferation	2	0-1 > 1-0-0-0-0
bS24D3#2	EH792596	Non-metastatic cells 1, protein (NM23A) expressed in (NME1)	Human	NM_198175.1	333/387 (86%)	cell proliferation	2	1-1-1-1 > 1-1-1
bS2B3#1	EH792598	Mitochondrion, [NADH dehydrogenase subunit 4L (ND4L)]	Porcine	AY334492	146/146 (100%)	metabolism	2	0-1-1-1-0-0-0
bS2B3#2	EH792599	Signal sequence receptor, gamma (SSR3)	Human	NM_007107	82/90 (91%)	other, unknown	2	0-1-1-1-0-0-0
bS2B4	EH792600	5'-nucleotidase domain containing 2 (NT5DC2)	Human	BC014550.1	120/130 (92%)	other, unknown	2	0-1-1-1-0-0-0
bS2B1	EH792597	Unknown				other, unknown	2	0-1-1-1-0-0-0
bS24D3#1	EH792595	EST	Porcine	BG835728	122/123 (99%)	other, unknown	2	1-1-1-1 > 1-1-1
bS3D7	EH792601	High mobility group AT-hook 2 (HMGA2)	Human	AF326972	198/235 (84%)	regulation of transcription	2	0-1 > 1 > 1-1-1-1
bS9C7	EH792614	Homo sapiens genomic DNA, chromosome 11q, clone:CTD-2011F17, [UV radiation resistance associated gene (UVRAG)]	Human	AP002340	58/67 (86%)	cell death, autophagy	3	0-0-0-0-1-1-1
qS24D2	EH792616	Tropomyosin 1, alpha (TPM1)	Porcine	X66274.1	373/376 (99%)	myofibrill assembly	3	0-1-1 < 1-1-1-1
bS15C2	EH792628	TIMP metallopeptidase inhibitor 2 (TIMP2)	Porcine	AF156030	146/178 (82%)	cell adhesion, ECM	4	0-0-1-1-1-1-1
bS19C1	EH792632	Immunoglobulin superfamily, member 1 (IGSF1)	Canine	XM_538173.1	351/391 (89%)	cell adhesion, ECM	4	0-1 < 1-1-1-1-1
bS3D8	EH792642	Actin, alpha, cardiac muscle (ACTC)	Human	NM_005159.3	172/184 (93%)	cell structure	4	0-1-1-1-1-1-1
bS14D2	EH792627	Cofilin 2, muscle (CFL2)	Human	NM_138638.1	128/139 (92%)	cell structure	4	0-0-1-1-1-1-1
bS20A3	EH792635	Myosin, heavy polypeptide 1, skeletal muscle, adult (MYH1)	Porcine	AB025262	237/245 (96%)	myofibrill assembly	4	0-0-1-1-1-1-1
bS1B3	EH792633	Troponin I type 2, skeletal, fast (TNNI2)	Porcine	NM_001032359	50/51 (98%)	myofibrill assembly	4	0-0-1-1-1-1-1
bS18B1	EH792631	Myopalladin (MYPN)	Canine	XM_546131.1	96/100 (96%)	myofibrill assembly	4	0-0-1-1-1-1-1
bS14C4	EH792626	Myosin, heavy polypeptide 3, skeletal muscle, embryonic (MYH3)	Bovine	AB090155.1	292/312 (93%)	myofibrill assembly	4	0-1 < 1-1-1-1-1
bS15C4	EH792629	Myosin, heavy polypeptide 2 (MYH2)	Porcine	NM_214136.1	249/270 (92%)	myofibrill assembly	4	0-0-1-1-1 > 1 > 1
qS4D7	EH792660	Titin (TTN)	Human	NM_003319.2	318/353 (90%)	myofibrill assembly	4	0-0-1 < 1 < 1-1-1
bS4B3	EH792643	Unknown				other, unknown	4	0-0-1-1-1-1-1
bS22B1#1	EH792636	EST [Chromosome 6 open reading frame 89 (C6orf89)]	Porcine	BP142021	147/151 (97%)	other, unknown	4	0-0-1-1-1-1-1
bS22B1#2	EH792637	Metastasis associated lung adenocarcinoma transcript 1 (MALAT1)	Human	NR_002819	204/240 (85%)	other, unknown	4	0-0-1-1-1-1-1
bS26B3	EH792640	EST	Porcine	BX918881	52/55 (94%)	other, unknown	4	0-0-1-1-1-1-1
bS22C2#1	EH792638	EST	Porcine	BQ599825	98/106 (92%)	other, unknown	4	0-1 < 1-1-1-1-1
bS22C2#2	EH792639	EST	Porcine	CB473363	90/105 (85%)	other, unknown	4	0-1 < 1-1-1-1-1
bS12D1	EH792624	EST	Porcine	CV866720	191/204 (93%)	other, unknown	4	0-1 < 1-1-1-1-1
bS17B3	EH792630	High-mobility group nucleosome binding domain 1 (HMGN1)	Canine	BX641076	292/308 (94%)	regulation of transcription	4	0-1 < 1 < 1 < 1 < 1 < 1
bS14C2	EH792625	DEP domain containing 5 (DEPDC5)	Human	BC057797.1	189/206 (91%)	signal transduction	4	0-0-1-1-1-1-1
bS20A2	EH792634	ATP synthase, H+ transporting, mitochondrial F1 complex, gamma polypeptide 1 (ATP5C1)	Bovine	XM_873667	177/195 (90%)	transport	4	0-0-1-1-1-1-1
bS2D1	EH792641	Hemoglobin, beta (HBB)	Porcine	X86791	191/197 (96%)	transport	4	0-1 < 1-1-1-1-1
qS20A1	EH792670	Mitochondrion, [NADH-ubiquinone oxidoreductase chain2 (ND2)]	Rabbit	AJ012536	237/240 (98%)	metabolism	5	1-1 > 1 > 1 > 1 > 1 > 1
qS15D1	EH792669	Ataxin 10 (ATXN10)	Porcine	AY550076	169/193 (87%)	other, unknown	5	1 > 1 > 1 > 1 > 1 > 1-1

^1^In square brackets the identity derived by cross-species megaBLAST search of the human genome sequence is indicated

^2 ^Binary coding of the differential display RT-PCR pattern (1-present, 0-absent)

**Table 2:** Summary of EST showing differences in expression during myogenesis between Pietrain and Duroc breeds of pigs

EST/Clone	Accession number	Identity^1^	Species	Accession number	Similarity	Function	Period group	Expression pattern^2^
qR15A1#2	EH792605	Androgen receptor N-terminal-interacting protein (ARNIP)	Human	AF247041	254/267 (95%)	metabolism	2	D-1-D-0-0-0-0
qR15A1#3	EH792606	NADP dependent leukotriene b4 12-hydroxydehydrogenase (LTB4DH)	Porcine	NM_214385	235/243 (96%)	metabolism	2	D-1-D-0-0-0-0
qR8A1	EH792608	Unknown				other, unknown	2	D-D-D-0-0-0-0
bR9C1	EH792594	Unknown				other, unknown	2	D-D-D-D-0-0-0
qR24C1	EH792607	Pan troglodytes chromosome 22 clone:RP43-009O02	Chimp	BS000013	92/112 (82%)	other, unknown	2	D-1-D-0-0-0-0
bR4D1	EH792593	Unknown				other, unknown	2	0-1-D-0-0-0-0
bR13B4	EH792592	GATA binding protein 3 (GATA3)	Porcine	DQ450901	189/193 (97%)	regulation of transcription	2	1-1-D-0-0-0-0
qR15A1#1	EH792604	DEAD (Asp-Glu-Ala-Asp) box polypeptide 17 (DDX17)	Human	NM_030881	230/259 (88%)	RNA/DNA metabolism	2	D-1-D-0-0-0-0
bR21D1	EH792610	Immunoglobulin superfamily, member 1 (IGSF1)	Canine	XM_538173	199/212 (93%)	cell adhesion, ECM	3	0-0-0-0-0-P-P
bR2B1	EH792611	Unknown				other, unknown	3	0-0-0-1-1-P-0
bR8B2	EH792613	Unknown				other, unknown	3	0-0-0-0-0-P-P
bR18D1	EH792609	Unknown				other, unknown	3	0-0-0-0-D-1-1
bR7D1	EH792612	RAB6 interacting protein 2 (RAB6IP2)	Human	NM_178037	268/287 (93%)	regulation of transcription	3	0-0-0-0-D-1-1
qR7B2	EH792615	Rap guanine nucleotide exchange factor (GEF) 5 (RAPGEF5)	Human	BC039203	62/73 (84%)	signal transduction	3	0-0-0-P-P-P-P
qR3B1	EH792655	EST [Laminin, beta 1 (LAMB1)]	Porcine	BE030491	171/174 (98%)	cell adhesion, ECM	4	0-P-P-P-1-1-1
qR4D2	EH792658	EST [Sarcospan (SSPN)]	Porcine	BX924101	469/475 (98%)	cell adhesion, ECM	4	0-P-P-1-1-1-1
qR19D1	EH792650	SPARC-like 1 (SPARCL1)	Human	NM_004684	198/236 (83%)	cell adhesion, ECM	4	0-0-P-P-P-P-P
qR22D1	EH792652	Tight junction protein 1 (TJP1)	Human	NM_003257	447/486 (91%)	cell adhesion, ECM	4	0-0-P-P-P-0-0
bR24D1	EH792623	Secreted phosphoprotein 1 (SPP1)	Porcine	AJ237667	125/126 (99%)	cell adhesion, ECM	4	0-0-D-D-D-D-D
qR14A1#2	EH792645	Spectrin repeat containing, nuclear envelope 2 (SYNE2)	Human	BC042134	88/100 (88%)	cell structure	4	0-0-1-D-D-D-D
qR1A1	EH792651	NADH dehydrogenase (ubiquinone) 1 alpha subcomplex 10 (NDUFA10)	Bovine	NM_176655	84/90 (93%)	metabolism	4	0-0-1-1-1-1-D
qR14A1#1	EH792644	Latexin (LXN)	Human	BC008438	133/151 (88%)	metabolism	4	0-0-1-D-D-D-D
bR13B8	EH792618	Nebulin-related anchoring protein (NRAP)	Porcine	DQ157553	199/200 (99%)	myofibrill assembly	4	0-P-P-P-P-P-0
bR24A1	EH792621	Unknown				other, unknown	4	0-P-P-P-P-P-P
qR4B1	EH792656	Unknown				other, unknown	4	0-P-P-P-1-1-1
qR24B1	EH792653	Metastasis associated lung adenocarcinoma transcript 1 (MALAT1)	Human	NR_002819	72/79 (91%)	other, unknown	4	0-0-P-P-1-1-1
qR25B3	EH792654	Unknown				other, unknown	4	0-0-P-P-P-P-P
qR5C1	EH792659	EST	Porcine	CJ014638	195/228 (85%)	other, unknown	4	0-0-P-P-1-1-P
qR15C1	EH792647	Unknown				other, unknown	4	0-D-P-P-P-0-0
qR19C1	EH792649	Unknown				other, unknown	4	0-0-P-P-1-1-1
qR4D1	EH792657	Unknown				other, unknown	4	0-P-P-P-1-1-1
bR15D1	EH792619	EST	Porcine	AJ956602	45/48 (93%)	other, unknown	4	0-0-P-P-P-P-P
bR22D2	EH792620	EST	Bovine	AW344601	49/52 (94%)	other, unknown	4	0-0-P-P-1-1-1
bR13B10	EH792617	EST [SMAD family member 7 (SMAD7)]	Bovine	DV922547	61/67 (91%)	regulation of transcription	4	0-P-P-0-P-P-P
bR24C2	EH792622	EST [SET binding protein 1 (SETBP1)]	Porcine	BP172194	369/373 (98%)	regulation of transcription	4	0-P-P-P-P-P-P
qR14C2	EH792646	Heterogeneous nuclear ribonucleoprotein D (HNRPD)	Murine	AK077409	265/279 (94%)	RNA/DNA metabolism	4	0-0-P-P-P-P-P
qR17B1	EH792648	Homo sapiens 12 BAC RP11-729I10 [ABCC9]	Human	AC008250	75/87 (86%)	transport	4	0-0-D-D-D-D-D
bR22B1	EH792663	Platelet-derived growth factor receptor, alpha polypeptide (PDGFRA)	Human	NM_006206	252/287 (87%)	cell proliferation	5	D-D-D-D-D-D-D
qR7B1	EH792668	SMC6 structural maintenance of chromosomes 6-like 1 (SMC6L1)	Canine	XM_532882	169/176 (96%)	other, unknown	5	D-1-P-P-1-1-1
bR26B1	EH792664	Unknown				other, unknown	5	P-P-P-P-1-P-1
qR10D1	EH792666	cDNA FLJ26539 fis [Bicaudal C homolog 1 (BICC1)]	Human	AK130049	65/71 (91%)	regulation of transcription	5	P-P-P-P-P-P-P
bR10D1	DQ631863	cDNA FLJ26539 fis [Bicaudal C homolog 1 (BICC1)]	Human	AK130049	65/71 (91%)	regulation of transcription	5	D-D-D-D-D-D-D
bR12D1	EH792661	EST	Porcine	AJ955354	51/51 (100%)	other, unknown	5	P-P-P-P-P-P-P
qR20D1	EH792667	EST	Porcine	DV230345	71/71 (100%)	other, unknown	5	P-P-P-P-P-P-P
bR2B2	EH792665	Jumonji domain containing 2A (JMJD2A)	Canine	XM_539647	278/305 (91%)	regulation of transcription	5	P-1-1-1-1-1-1
bR13A3	EH792662	Poly(A) polymerase gamma (PAPOLG)	Bovine	XM_607511	217/229 (94%)	RNA/DNA metabolism	5	D-D-D-D-D-D-D

^1^In square brackets the identity derived by cross-species megaBLAST search of the human genome sequence is indicated

^2^Binary coding of the differential display RT-PCR pattern (1-present in both breeds, 0-absent in both breeds, P-Present/More intense in Pietrain, D- Present/More intense in Duroc)

The most frequent functional categories represented genes encoding myofibrillar genes (8 genes: *MYH3*, *MYH1*, *TNNI2*, *MYPN*, *MYH2*, *TTN*, *NRAP*, *TPM1*), genes involved in cell adhesion, cell-cell signaling and extracellular matrix synthesis/remodeling (8 genes: *SDC3*, *LAMB1*, *SSPN*, *IGSF1*, *SPARCL1*, *SPP1*, *TIMP2*, *TJP1*), genes regulating gene expression (8 genes: *BICC1*, *GATA3*, *HMGA2*, *HMGN1*, *JMJD2A*, *RAB6IP2*, *SETBP1*, *SMAD7*) and metabolism genes (8 genes: *ARNIP*, *GLUD1*,*LTB4DH*, *LXN*, *ND2, ND4L*, *NDUFA10*, *RPL37*). The myofibrillar genes and genes involved in cell adhesion, cell-cell signaling and extracellular matrix synthesis/remodeling were almost exclusively represented in the period 4 group (i.e. they were expressed from 21–35 dpc onward).

Another functional group enriched in the period 4 group were cytoskeletal genes related to assembly of actin filaments (3 genes: *ACTC*, *CFL2*, *SYNE2*). The remaining functional groups showed no obvious enrichment in a specific period group.

Regarding the breed-associated cDNA fragments, 28 were present/more intense in Pietrain and 15 were present/more intense in Duroc (Table 2). Genes related to cell adhesion, cell-cell signaling and extracellular matrix synthesis/remodeling and those from period group 4 tended to be enriched among the Pietrain differentials and genes related to metabolism and those from period group 2 tended to be enriched among the Duroc differentials.

### Analysis of expression profiles of reference genes

**Figure 1:**
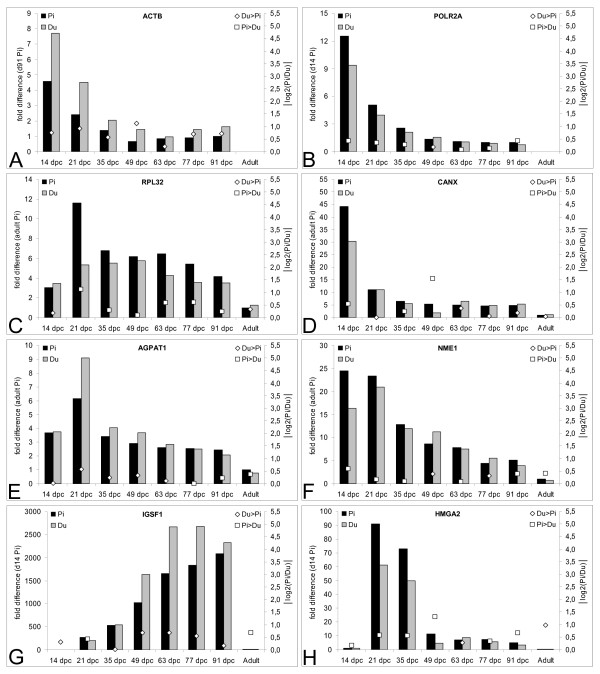
**Expression of reference genes (A-E) and genes showing stage-associated differential display RT-PCR patterns (F-H) obtained by qPCR**. Histograms show normalized relative gene expression in Pietrain and Duroc embryos/*Musculus longissimus dorsi *at seven prenatal stages and in the adults (primary y-axis). For some genes data on expression in adults is missing. Open boxes and diamonds show absolute values of the Pietrain vs. Duroc expression ratios at each stage (log2(Pi/Du)). Open diamonds indicate higher expression in Duroc compared to Pietrain individuals and open boxes indicate the reverse situation (secondary y-axis).

The use of reference genes for normalization of real-time quantitative PCR (qPCR) data requires identification of genes which show constant expression under given experimental conditions. We analyzed expression profile of five potential reference genes, three (*ACTB*, *RPL32*, *POLR2A*) widely used and described [15,16], and two (*AGPAT1 *involved in phospholipid metabolism and *CANX *involved in protein folding) showing stable expression in microarray studies comparing expression signatures of different tissues and/or developmental stages [17,18]. Not unexpectedly, considering the major changes in the cell type composition and the structure of the tissue samples, none of the five genes turned out to be expressed at constant levels across the seven prenatal stages (Figure 1). Expression of *ACTB*, *POLR2A*, *CANX *showed steady downregulation from 14 dpc onwards, whereas expression of *RPL32 *and *AGPAT1 *peaked at 21 dpc and was downregulated thereafter. All three reference genes analyzed in adult muscle *(RPL32*, *AGPAT1*, *CANX*) were downregulated compared to prenatal stages. Therefore we calculated a normalization factor based on the five reference genes (three for adult muscle) within stages, thus accounting for technical variation between breeds but not between stages.

### Validation of stage- and/or breed-associated differential expression of individual genes

In previous expression studies performed in our laboratory using differential display RT-PCR the incidence of false positives was low with about 23% [19], however, in other studies employing differential display RT-PCR the rate of false positives reached as much as ~80% in some cases [20]. Therefore expression patterns of thirteen genes showing stage- and/or breed-associated differential expression were validated by qPCR. We focused the validation mainly on the breed-associated genes because they represent primary candidates for finding the source of variation in muscularity and meat quality in pigs. All genes were selected so that (1) different expression patterns and/or (2) different functional pathways were represented.

Three stage-associated genes, namely *NME*, *IGSF1 *and *HMGA*, were validated.

Expression of *NME1 *showed steady downregulation from 21 dpc onward (Figure 1F).

Expression of *IGSF1 *was low/absent at 14 dpc, increased dramatically until 63 dpc, stayed at similar level until 91 dpc and was downregulated in adult muscle nearly to the starting level at 14 dpc (Figure 1G). At all stages the differences in expression of *NME1 *and *IGSF1 *between Pietrain and Duroc were lower than twofold.

Expression of *HMGA2 *was barely detectable at 14 dpc, reached maximum at 21–35 dpc, decreased substantially at 49 dpc, stayed at similar low level until 91 dpc and was essentially shut down in adult muscle (Figure 1H). The sensitive qPCR revealed a ~2.5-fold higher expression of *HMGA2 *in Pietrain at 49 dpc, not detected by differential display RT-PCR.

The expression patterns of ten breed-associated genes and EST were validated.

**Figure 2:**
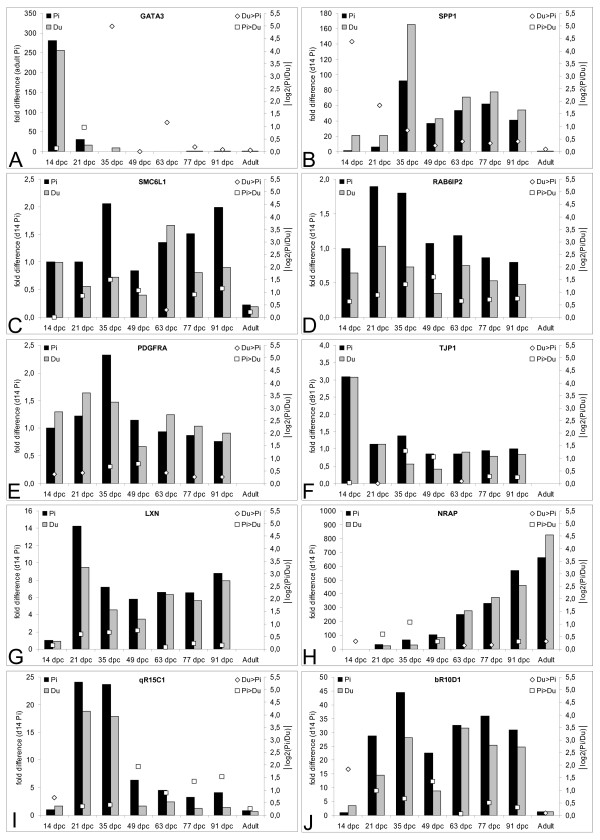
**Expression of ten genes showing breed-associated differences in differential display RT-PCR patterns obtained by qPCR**. Histograms show normalized relative gene expression in Pietrain and Duroc embryos/*Musculus longissimus dorsi *at seven prenatal stages and in the adults (primary y-axis). For some genes data on expression in adults is missing. Open boxes and diamonds show absolute values of the Pietrain vs. Duroc expression ratios at each stage (log2(Pi/Du)). Open diamonds indicate higher expression in Duroc compared to Pietrain individuals and open boxes indicate the reverse situation (secondary y-axis).

Expression of *GATA3 *as revealed by qPCR was highest at 14 dpc (Figure 2A). At 21 dpc the expression decreased, with more pronounced downregulation in Duroc embryos. In agreement with the results of differential display RT-PCR the expression lasted in Duroc embryos/fetuses until 49 dpc whereas in Pietrain embryos/fetuses the expression ceased already at 35 dpc.

The EST bR24D1, that was found in Duroc from 35 dpc onward only, was homologous to intron 6 of the *SPP1 *gene. Since we used total RNA for differential display RT-PCR we reasoned that this EST was derived from heteronuclear RNA (hnRNA) and thereby reflected higher active transcription of *SPP1 *in Duroc. QPCR using exon specific primers showed that expression of *SPP1 *peaked at 35 and 77 dpc, i.e. at the time-points of the two myogenic waves, and confirmed consistently higher expression of *SPP1 *in Duroc at all seven prenatal stages (Figure 2B). The difference was highest at 14 dpc (~20-fold), afterwards gradually decreased until 49 dpc (~3.6-fold to ~1.3-fold) and remained at similar magnitude until 91 dpc. In adult muscle no differences were found.

The *SMC6L1 *gene was expressed throughout myogenesis as shown consistently by both, differential display RT-PCR and qPCR (Figure 2C). QPCR revealed more than twofold upregulation of *SMC6L1 *at 35 and 49 dpc in Pietrain in agreement with differential display RT-PCR and additionally at 91 dpc, but no difference was found at 14 dpc.

For the remaining breed-associated EST/genes no clear relationship between profiles obtained using differential display RT-PCR and qPCR respectively could be established.

The expression profile of *RAB6IP2 *showed two peaks, one higher at 21–35 dpc and another at 63 dpc, i.e. at the onset of the two waves of myofiber formation. Expression of *RAB6IP2 *was consistently upregulated (~1.5 to ~3-fold) throughout myogenesis in Pietrain compared to Duroc with the most pronounced differences at 35–49 dpc (~2.5 to ~3-fold; Figure 2D).

The expression pattern of the EST bR22B1, homologous to *PDGFRA*, obtained by differential display RT-PCR suggested upregulation of *PDGFRA *in Duroc compared to Pietrain embryos/fetuses. Indeed such upregulation was found by qPCR at all stages but 35 and 49 dpc when the situation was reversed (Figure 2E). The 1.2–1.4-fold upregulation of PDGFRA detected by qPCR in Duroc correlated poorly with the results of differential display RT-PCR. However, qPCR revealed a striking difference in the expression pattern between Pietrain and Duroc. In Pietrain the expression of *PDGFRA *gene showed a peak at 35 dpc whereas in Duroc two peaks existed, one at 21 dpc and another at 63 dpc.

For *TJP1 *both, differential display RT-PCR and qPCR indicated upregulation (≥2-fold) at 35 and 49 dpc in Pietrain compared to Duroc (Figure 2F). No difference at 63 dpc could be shown by qPCR. The expression of *TJP1 *was highest at 14 dpc and in Duroc continuously decreased until 49 dpc. In Pietrain a small peak at 35 dpc was observed. From 63 dpc onward the expression stayed at a similar level until 91 dpc in both breeds.

The expression profile of *LXN *as revealed by qPCR (Figure 2G) was similar to the expression profiles of *RPL32 *and *AGPAT1 *(Figure 1C and 1E). The differences in the expression of *LXN *between breeds were less than twofold, but especially in the early stages 21–49 dpc the expression was consistently higher in Pietrain.

The expression of *NRAP*, as revealed by qPCR, was barely detectable at 14 dpc and continuously increased in the developing muscle until adult age (Figure 2H). At 35 dpc the expression of *NRAP *was ~2-fold higher in Pietrain compared to Duroc fetuses.

The expression profile of the EST qR15C1 obtained using qPCR resembled those of the *HMGA2 *gene (Figure 2I). A peak was observed at 21–35 dpc and from day 49 dpc onward the expression was maintained at low level; in Pietrain about 2–4-fold higher compared to Duroc. In adult muscle the expression was essentially switched off. The temporal expression profile of the EST qR15C1 indicated that the transcript might be involved in early development. Unfortunately, the sequence of qR15C1 was very short and showed no similarity with transcripts of known genes. We took advantage of the ongoing sequencing of the porcine genome and retrieved a trace sequence [NCBI Trace Archiv:Ti 784825315] overlapping the EST. Using this trace we were able to map the EST qR15C1 to intron 4 of the human kinesin family member 26B (*KIF26B*) gene (Hsa1q44, 82% identity over 93 bp at 243749890 bp). Thus qR15C1 may represent a so far undetected exon of *KIF26B*.

Sequencing of the two comigrating cDNA fragments bR10D1 and qR10D1 revealed that they represent the same transcript differing only in a 3 bp insertion/deletion and that the differences between breeds obtained using differential display RT-PCR were based on different frequencies of the insertion/deletion in Duroc and Pietrain. Nevertheless qPCR revealed that besides allelic variation the transcript represented by the EST bR10D1/qR10D1 differed also in its abundance between the two breeds (Figure 2J). In the early Duroc embryos at 14 dpc the expression was about 4-fold higher compared to Pietrain and the situation was reversed during the first wave of myogenesis at 21–49 dpc, when the expression was about 1.5 to 2.5-fold higher in Pietrain embryos/fetuses. The expression profile was very similar to that of *SPP1*, i.e. it peaked at 35 dpc and 77 dpc respectively.

Taken together, qPCR confirmed the differential display RT-PCR derived expression patterns for all three genes showing stage-associated differential expression and three out of ten genes with breed-associated differential expression (overall incidence of false positives ~50%). Furthermore, five of the seven breed-associated genes, whose differential display expression pattern could not be reflected in detail by qPCR, showed more than twofold differences in expression between breeds on at least one stage.

## Discussion

### Identification of genes/networks expressed during skeletal muscle development in pigs

Myogenesis in mammals is a multistep process that involves commitment of pluripotent mesodermal cells in somites to the myogenic lineage, followed by proliferation/migration of myoblasts and two waves of myoblast fusion resulting in the formation of two morphologically/functionally distinct types of myotubes.

In the present study we performed expression profiling of skeletal muscle development in the pig using embryonic and fetal *musculus longissimus dorsi *tissue at seven stages covering the whole myogenesis from somitogenesis to maturation of the myotubes into myofibers. Recently Te Pas et al [11,13] reported results of a similar study analyzing the same breeds and developmental stages using an application specific microarray focused on genes related to myogenic regulatory factors, energy metabolism and structural genes. Here we provide complementary data generated using differential display RT-PCR as a hypothesis free, open system. We identified 85 transcripts expressed at one or more of these key stages, evidencing their potential involvement in myogenesis. These transcripts represented 52 genes from various functional classes and 33 anonymous EST. Several of them were either not yet cloned in the pig (~30%), or are completely novel (~20%). The identified genes and networks and their relationship with myogenesis are discussed below.

At early developmental stages (14 and 21 dpc) it was not feasible to dissect structures giving rise to muscle and so expression profiles of some of the transcripts may have been influenced by the heterogeneous and changing cellular composition. For ~50% of the transcripts the expression profiles derived by differential display RT-PCR and qPCR are not fully compatible. In the case of stage-associated EST/genes the consistency of expression patterns in both breeds provides biological replication and thus underscores the reproducibility of the results. On the other hand, the differences in differential display RT-PCR and qPCR profiles of breed-associated EST/genes, especially of those displayed in binary fashion, may have resulted from different sensitivity of the two methods (e.g. for bR22B1/*PDGFRA *gene) and polymorphisms in some of the EST (e.g. bR10D1 and qR10D1). Because for qPCR very short amplicons are usually designed to achieve high amplification efficiency it is also possible that in some cases the differential display cDNA fragment and the qPCR amplicon represented different splice forms of the same gene and thus show different profiles.

The differential display patterns revealed on average some 5 bands representing either genes with stage- and/or breed-specific expression that were further assigned to groups according to their temporal expression pattern and to categories of biological functions. A subset of genes representing these categories was analysed further by qPCR. These are discussed in the following paragraphs.

### Genes involved in the regulation of transcription

Members of the myogenic regulatory factor (MRF) and myocyte enhancer factor 2 (MEF2) families of transcription factors play a central role in the regulation of myogenic determination and differentiation; however, their expression during porcine myogenesis changes only marginally [13]. Recently Blais et al [21] identified an extensive network of genes regulating transcription and chromatin architecture that propagate and amplify the myogenic signal initiated by the MRFs *in vitro*. Among the genes we identified, those regulating transcription represented one of the most abundantly detected functional classes, underscoring the important role of transcriptional regulation in the control of myogenesis. For the majority of genes identified, their precise role in this process is largely unknown, but because they were expressed in various 'period' groups they probably have diverse functions. The architectural transcription factor *HMGA2*, for example, was recently shown to promote myogenic differentiation *in vitro *[22]. However, the EST bS3D7 does not show homology to known cDNA sequence of *HMGA2 *but to a sequence segment in the intron 3 of the human *HMGA2 *[GenBank:AY387666], that was shown to be transcribed in uterine leiomas. Thus, EST bS3D7 probably represents a novel exon of porcine *HMGA2*. Several studies in humans point to the existence of alternative exons in intron 3 of *HMGA2 *with a complex splicing pattern [23]. The porcine EST harbours sequence features that support the hypothesis of an alternative exon – an open reading frame (ORF) with a stop codon, a polyadenylation signal and a poly-A tail. Expression of this exon would generate a HMGA2 protein with a truncated C-terminal region and lacking the acidic tail. Intriguingly, overexpression of a carboxyl-terminally truncated HMGA2 form in embryonic stem (ES) cells resulted in enhanced myogenic differentiation compared to wild type HMGA2 cells [22]. Thus the putative novel exon of porcine *HMGA2 *may play a specific function in myogenesis.

Another gene of this functional class, *RAB6IP2*, showed consistent upregulation at all seven stages in Pietrain compared to Duroc samples. The function of *RAB6IP2 *is poorly understood. Recently, Sigala et al [24] proposed RAB6IP2 as a part of the IkappaB kinase complex, involved in activation of the transcription factor NF-kappaB. NF-kappaB in turn inhibits myogenic differentiation and stimulates proliferation of myoblasts by activation of cyclin D1 transcription and suppression of MYOD [25]. Hence upregulation of *RAB6IP2 *may be an indicator for delayed myogenic differentiation via NF-kappaB in Pietrain.

### Genes involved in the control of cell proliferation

Proliferation of myoblasts is stimulated by a variety of secreted peptide growth factors including insulin-like growth factors (IGFs) and platelet derived growth factors (PDGFs). These usually have reverse effect on myogenic differentiation with the exception of IGFs that stimulate both proliferation and differentiation of myoblasts [26]. We found a new and largely uncharacterized member of the IGF system, *IGFBPL1*, among genes expressed during the first wave of myogenesis. The IGF binding proteins (IGFBP) serve as modulators of the effects of IGFs. IGFBPL1 shows structural and functional similarity to IGFBP7 [27], that specifies the proliferative response to IGFs in myogenesis [28]. Taken together the expression pattern and similarity to IGFBP7 it implies that IGFBPL1 may also serve as a factor stimulating proliferation and/or inhibiting differentiation of embryonic myoblasts.

The expression profile of *PDGFRA *showed distinct differences between Pietrain and Duroc at early stages of myogenesis, with Pietrain showing a more pronounced but delayed peak. *PDGFRA *plays a vital role in the early myogenesis as evidenced by the *PDGFRA *null mouse showing impaired myotome formation [29] and (nearly) complete absence of muscles of the back [30].

### Cell adhesion and extracellular matrix genes

Signals generated by adhesive interactions between myoblasts and between myoblasts and the extracellular matrix represent another essential part of the molecular network governing myogenic differentiation besides secreted factors and transcription factors [31,32]. Furthermore, cell adhesion and extracellular matrix molecules serve a structural role in myoblast fusion and migration, and provide physical support for the developing myofibers. Consistently, genes involved in cell adhesion, cell-cell signaling, and extracellular matrix synthesis and remodeling were abundantly represented and were enriched in the period 4 group, i.e. they were expressed from 21–35 dpc onward. Several of these, like *IGSF1*, were not yet associated with myogenesis. *IGSF1 *encodes a member of the immunoglobulin superfamily (IgSF) of cell surface proteins mediating cell adhesion and recognition. Diverse IgSF members have been implicated in cell-contact-based regulation of myogenesis [31]. The increasing expression of *IGSF1 *towards later stages suggests that it might be involved in the second wave of myogenesis.

Expression of *SPP1 *shows a more regulated profile with peaks at the two respective myogenic waves and consistent upregulation in Duroc compared to Pietrain, especially at the early stages. SPP1 is multifunctional matricellular protein mediating cell-adhesion and cellular signaling via integrin and CD44 receptors. Several lines of evidence indicate that SPP1 is involved in myogenesis. *SPP1 *is a target of MYOD and MYF5 [33] and was shown to be expressed *in vitro *in myoblasts and myotubes, with higher levels in the later [34]. Moreover *SPP1 *is downregulated in the BC3H1 cell line, which is myogenic but do not terminally differentiate, compared to the myogenic C2C12 cell line, which differentiate normally [35]. Considering our *in vivo *and the *in vitro *evidences for an association between *SPP1 *expression and myogenic differentiation it appears that upregulation of *SPP1 *in Duroc may be a sign of accelerated myogenesis in this breed compared to Pietrain.

### Differential gene expression as an indicator of cellular reorganization during myogenesis

The abundantly identified myofibrillar, cytoskeletal, and metabolism genes are indicators of the massive structural and functional remodeling occurring during the development of skeletal muscle at the cellular level, including for example formation of the contractile apparatus. Another indicator of the ongoing reorganization of cell and tissue architecture might be the expression of the autophagy genes *ATG3 *and *UVRAG *that we detected during the first and second wave of myogenesis, respectively. Autophagy is a process of bulk degradation of cytoplasmatic components that occurs in a wide range of eukaryotic organisms and in multiple different cell types during nutrient and growth factor deprivation, cellular and tissue remodeling, and cell death. The importance of autophagy for normal development is emphasized by frequent observation of dysregulation of autophagy in cancer [36]. To our best knowledge there are no reports describing autophagy and its exact role during myogenesis.

### Novel transcripts

Of the 85 EST generated, 33 (~39%) showed no appreciable similarity to transcripts of known genes. Remarkably, these EST were enriched among the breed-associated cDNA-fragments. This finding may indicate that these EST represent alternative exons with specific temporo-spatial expression, whose alternative usage may generate transcript diversity and ultimately phenotypic (i.e. breed) diversity [37]. The expression profiles of several of these EST suggest involvement of the corresponding genes in myogenesis or prenatal development in general. One interesting example is the EST qR15C1, whose expression is upregulated at 21 and 35 dpc. The EST qR15C1 probably represents a novel exon of *KIF26B *since comparative genome analysis showed similarity to intron 4 of the human ortholog. Moreover, *KIF26B *showed a similar expression profile as it is preferentially expressed in limb buds and, most notably, in the precursors of hypaxial muscles in somites of mouse embryos [38].

### Implications for the understanding of the genetic control of muscle growth

Genetic and epigenetic factors controlling proliferation, differentiation and cell death affect the number of myoblasts available for myofiber formation and thus are important determinants of muscle growth. We identified several novel genes that may be involved in these processes, e.g. *IGFBPL1*, during myogenesis. Particularly interesting are those genes that show differential expression between the two breeds differing in muscularity. Taken together, the breed-associated differences in the expression of *RAB6IP2*, *PDGFRA *and *SPP1 *suggest that myogenesis is delayed especially during the first wave in Pietrain, the more muscular of the two breeds, allowing the generation of a larger pool of muscle precursor cells. This finding is in accordance with results of Te Pas et al [11], who found that myogenesis genes show lower expression in Pietrain compared to Duroc at early stages, probably delaying formation of primary myotubes. The number and size of primary myotubes are intrinsic factors affecting secondary fiber number. In contrast to secondary myotubes, whose number is sensitive to exogenous factors, e.g. nutrition, the number of primary myotubes is genetically programmed [39]. Thus genetic selection may impinge on the first wave of myogenesis, promoting genes active at this stage as primary candidates for manipulation of muscle growth.

## Conclusion

The present study revealed several genes differentially expressed during skeletal muscle development of domestic pig that were not yet associated with myogenesis and thus provide novel insights into molecular pathways employed in mammalian myogenesis (e.g. the autophagy pathway) and a foundation for future functional studies. Genes that exhibited differences between the divergent breeds represent candidate genes for muscle growth and structure. Indeed several of the identified genes map to known porcine QTL regions affecting muscle growth and/or structure and their DNA variation is associated with variation in traits related to muscle deposition [40,41].

## Methods

### Tissue sampling and RNA extraction

To obtain embryos/fetuses 99 Pietrain and 105 Duroc sows respectively (12 to 17 sows per stage) were artificially inseminated with semen from purebred sires and slaughtered at 14, 21, 35, 49, 63, 77 and 91 days *post conception *(dpc). Immediately after exsanguination of the sows the uteri were recovered and the embryos/fetuses were quickly removed, weighted (35 dpc onward), and dissected. Samples were frozen in liquid nitrogen and stored at -80°C. To harvest the embryos at 14 dpc each uterus horn was flushed with PBS, i.e. at this stage each sample represented the whole litter of a sow. At 21 dpc whole embryos were collected and for RNA isolation the dorsolateral part was used. At 35 dpc precursor tissue of the back muscles was dissected from the area along the spine. From day 49 dpc onward *Musculus longissimus dorsi *(Mld) tissue samples were dissected. In total about 1000 samples from each of the two breeds were collected. In addition Mld samples from each three Pietrain and Duroc sows respectively were obtained. All handling of the animals was done in accordance with German law for the Protection of Animals and was approved by the animal welfare protection commission of the University of Bonn and the responsible veterinary authority.

Before 49 dpc the sexual characteristics were not visible therefore gender of the embryos and fetuses at 21 and 35 dpc was determined using a PCR-based method according to [42] with minor modifications. Briefly, a fragment of the sex-determining region Y located on the Y chromosome was amplified using the primers SRYB-3 and SRYB-5 along with the autosomal locus STS-Bo1 [43] serving as an internal control. The cycling parameters used were as follows: 95°C for 3 min; 36 cycles of 95°C for 10 s, 62°C for 30 s, 72°C for 30 s; and 72°C for 5 min.

Total RNA was isolated using TRI Reagent (Sigma, Taufkirchen, Germany) according to the manufacturer's protocol. After DNaseI treatment (Promega, Mannheim, Germany) the RNA was cleaned up using the RNeasy Kit (Qiagen, Hilden, Germany), the quantity was spectrophotometrically determined and the integrity checked by electrophoresis of 500 ng RNA on ethidium bromide stained 1% denaturing agarose gels. In addition absence of DNA contamination was checked using the RNA as a template in a PCR amplifying a fragment of the glyceraldehyde-3-phosphate dehydrogenase (*GAPDH*) gene.

### Differential display RT-PCR

To minimize the negative impact of individual variation in the gene expression on the expression profiles RNA pools were used for each stage and breed, i.e. 14 RNA pools were made (2 breeds × 7 stages). With the exception of the embryo samples at 14 dpc the pools were prepared by mixing of equal amounts of RNA from 10 individuals per breed, one male and one female sibling from 5 litters per stage. The individuals were selected according to their age and weight/size to be representative for their breeds at the chosen developmental stages. For the embryos at 14 dpc the individuals were already pooled during the harvesting, therefore the RNA pools were set up from uterus flushes of 5 sows per breed.

First-strand cDNA was synthesized using SuperScriptII MMLV reverse transcriptase (Invitrogen, Karlsruhe, Germany) and each of the oligo (dT)_11_CG, (dT)_11_GC, (dT)_11_GG und (dT)_11_CC primers. Differential display RT-PCR was performed according to [19] using random decamer primers developed by Bauer et al. [44]. In total 88 primer combinations were used to generate differential display profiles. Each differential display RT-PCR was performed in duplicate to minimize false positives and the PCR products from both reactions were loaded in parallel on 5 % denaturing polyacrylamide gels, separated by electrophoresis for 4.5 hours and visualized by silver staining.

### Isolation, sequencing and annotation of the differentially displayed cDNA fragments

Selected cDNA fragments with breed or stage-associated appearance were excised using sterile needles, eluted in 2 × PCR buffer at 4°C overnight, precipitated using linear acrylamide as carrier, eluted in 20 μl of PCR grade H_2_O, and reamplified using the same PCR conditions as for differential display RT-PCR but with the cycle number increased to 50. Presence of the target fragment was checked by running an aliquot of the reamplification on 5 % denaturing PAA gels. The reamplified products were subsequently purified on 1 % agarose gels and cloned using either the pGEM-T (Promega) or the pCR2.1 (Invitrogen) vector. Following an insert amplification using M13 primers at least two clones per fragment were sequenced on Licor 4200 sequencer using the SequiTherm EXCEL Kit (Biozym, Hess. Oldendorf, Germany) and Sp6 and T7 primers. The identity of the expressed sequence tags (EST) was first determined by BLASTN search against the nonredundant and EST GenBank repositories (BLASTN identities > 80%). For EST for which sequence identity was found only to anonymous EST or genomic clones the corresponding gene was attempted to be identified by cross-species megaBLAST search against the human genome by taking advantage of the high homology between pig and human genome sequences. The EST for which the gene identity could be derived were assigned to functional categories based on GO annotation where available or inferred from the available literature.

### Real-time quantitative PCR

Real-time quantitative PCR (qPCR) was employed for validation of the expression patterns of selected EST.

Primers and amplicons shown in Table 3, were designed using the Primer Express v2.0 (Applied Biosystems, Weiterstadt, Germany). For all target EST/genes with the exception of the *SPP1 *gene, the *LXN *gene and the EST qR15C1 the primers were designed from sequences obtained in the present study. The sequences of the EST qR14A1#1 (*LXN*) and qR15C1 were too short to design primers suitable for qPCR therefore overlapping sequences, identified by BLASTN search of available porcine sequences, were used for primer/amplicon design (Table 3). For the *SPP1 *gene the porcine reference sequence was used (Table 3). Sequences of the *ACTB*, *CANX*, *AGPAT1 *and *RPL32 *genes were retrieved from GenBank by BLASTN search using the corresponding human gene as reference. The primers used for quantification of the *POLR2A *gene were kindly provided by Dr. Manfred Mielenz, Institute of Physiology, Biochemistry and Animal Hygiene, University of Bonn.

**Table 3:** Information on primers used for qPCR

Gene/EST	Source	Primer Sequence	Ta (°C)	Amplicon size (bp)
*ACTB*^1^	AJ312193	GAGAAGCTCTGCTACGTCGC	59	231
		CCTGATGTCCACGTCGCACT		
*POLR2A*^1^	DT324622	GAAGGGGGAGAGACAAACTG	60	86
		GGGAGGAAGAAGAAAAAGGG		
*RPL32*^1^	NM_001001636	AGCCCAAGATCGTCAAAAAG	55	165
		TGTTGCTCCCATAACCAATG		
*AGPAT1*^1^	AL773562	AGGACGCAACGTCGAGAACA	60	110
		GTGAGGGAGGGAAGTGGTGAG		
*CANX*^1^	AJ653783	CAATGATGGATGGGGTCTGAA	60	135
		AACACAGGTAATGCCACAGTCAA		
NME1^2^	EH792596	TGTGGAGAGCGCAGAGAAAGA	58	143
		GGGAGAGAGGAGAAATGGAATGG		
*IGSF1*^2^	EH792632	GAGTCCACCCCATCTACTGTTCC	60	60
		AAATCCCCTTGACCCATCTCA		
*HMGA2*^2^	EH792601	AAGGAGGCAGAAGCAGAATGA	59	74
		TGGAGACCCTCAGAGACAAGAA		
*GATA3*^3^	EH792592	CTTAGGGAAGATGAGTCTGAATGG	59	125
		TTTTGAAGGCAGAAAGCGAAG		
*SPP1*^3^	X16575	TTGCTAAAGCCTGACCCATCT	60	145
		CGTCGTCCACATCGTCTGTT		
*SMC6L1*^3^	EH792668	TGAGGCAGTATCAAGAAGCAAAAG	58	189
		AACAACAGCAACAAAAGAGCCAA		
*RAB6IP2*^3^	EH792612	GGATGTGAAGGAGCGGAAAG	60	121
		AGCCTGCAATGATTTGACTCG		
*PDGFRA*^3^	EH792663	CAGGCAGGTTGGAGGGAGAT	60	101
		AAGTTGCGGAGGTTGGATTCT		
*TJP1*^3^	EH792652	CTGGGCTCTTGGCTTGCTATTC	60	130
		CTCCTCCTGCCGTTTTTGG		
*LXN*^3^	CB477731	CAAGCAAGTGCAAAGAAATGATG	60	151
		TGGCAGACGGCTGTTATGTT		
*NRAP*^3^	EH792618	AAGTGAGGCAGTCTCCAGAGG	60	86
		CATATCCCAGTGAAACACCGAT		
*qR15C1*^3^	Ti784825315	ACAGTGAGAGCGAGCGTGATG	60	158
		TGCTTTCCCTTTATCGGAGG		
*bR10D1*^3^	DQ631863	GCTACACATTCAGCACAGAGTAAGA	60	126
		CTGGGGAAAGACTCCAAAAGA		

^1^Reference gene

^2^Gene showing stage-associated differential expression as revealed by differential display RT-PCR

^3^Gene showing breed-associated differential expression as revealed by differential display RT-PCR

Template first strand cDNA for validation was synthesized from the original 14 RNA pools used for the differential display RT-PCR using SuperScriptIII MMLV reverse transcriptase (Invitrogen) in a reaction containing 1 μg RNA and oligo (dT)_11_VN primer according to the manufacturer's protocol. In addition cDNA was synthesized using RNA pools from Mld tissue of 3 adult sows per breed as template.

Absolute quantification of the expression was performed on an ABI Prism 7000 SDS Instrument SDS v1.1 using the SYBR Green JumpStart Taq ReadyMix (Sigma) or, for the GATA3 and the RPL32 genes, on a LightCycler 1.0 System using the LightCycler FastStart DNA Master SYBR^plus ^Green I (Roche Diagnostics, Mannheim, Germany). All reactions were performed in duplicate with standard deviation between replicates kept below fifty percent. On the ABI Prism 7000 SDS Instrument a two-step PCR was performed with an initial denaturation at 95°C for 10 min and 45 cycles of 95°C for 15 s for denaturation and one-minute annealing/extension at optimized temperature (Ta, Table 3). The fluorescence was acquisited at the end of each cycle at a fixed temperature of 60°C. On the LightCycler instrument the qPCR consisted of an initial denaturation step at 95°C for 10 min and 45 cycles consisting of 95°C for 10s, optimal Ta for 15 s and extension at 72°C for 15 s. The fluorescence was acquisited at the end of the extension step. After completion of the qPCR on both instruments melting curve analysis and afterwards agarose gel electrophoresis were performed to confirm specificity of the amplification.

For all assays threshold cycles were converted to copy numbers using a standard curve generated by amplifying serial dilutions of an external plasmid standard (10^7 ^- 10^1 ^copies). To account for variation in RNA input and efficiency of reverse transcription the calculated copy numbers were normalized by dividing with a normalization factor derived from the expression of the five (three for adult stage) reference genes. First, for each reference gene a correction factor was calculated within stage and breed by dividing the quantity obtained for the corresponding breed by the mean quantity of the two breeds at that specific stage. The normalizing factor was then calculated for each breed and stage based on the geometric mean of the five (three) correction factors.

## Competing interests

The author(s) declares that there are no competing interests.

## Authors' contributions

EM participated in sampling, carried out differential display RT-PCR analysis and data analysis and wrote the manuscript. MM carried out sequencing, sequence annotation and qPCR analysis. SP provided input to design of the study, participated in sampling and assisted drafting the manuscript and revising it critically for scientific content. KS made contribution to the study design. KW significantly contributed to the concept, design and coordination of the study and in drafting the manuscript. All authors read and approved the final manuscript.
